# Genome Wide Association Study with Imputed Whole Genome Sequence Data Identifies a 431 kb Risk Haplotype on CFA18 for Congenital Laryngeal Paralysis in Alaskan Sled Dogs

**DOI:** 10.3390/genes13101808

**Published:** 2022-10-06

**Authors:** Krishnamoorthy Srikanth, Dirsko J. F. von Pfeil, Bryden J. Stanley, Caroline Griffitts, Heather J. Huson

**Affiliations:** 1 Department of Animal Science, College of Agriculture and Life Science, Cornell University, Ithaca, NY 14850, USA; 2 Small Animal Surgery Locum, PLLC, Dallas, TX 75201, USA; 3 Department of Small Animal Clinical Sciences, Michigan State University, East Lansing, MI 48824, USA; 4 The Travelling Vet LLC, Loveland, CO 80537, USA

**Keywords:** congenital laryngeal paralysis, Alaskan sled dog, Alaskan husky, GWAS, WGS, risk haplotype, CFA18, *ALX4*, *EXT2*, *TSPAN53I11*

## Abstract

Congenital laryngeal paralysis (CLP) is an inherited disorder that affects the ability of the dog to exercise and precludes it from functioning as a working sled dog. Though CLP is known to occur in Alaskan sled dogs (ASDs) since 1986, the genetic mutation underlying the disease has not been reported. Using a genome-wide association study (GWAS), we identified a 708 kb region on CFA 18 harboring 226 SNPs to be significantly associated with CLP. The significant SNPs explained 47.06% of the heritability of CLP. We narrowed the region to 431 kb through autozygosity mapping and found 18 of the 20 cases to be homozygous for the risk haplotype. Whole genome sequencing of two cases and a control ASD, and comparison with the genome of 657 dogs from various breeds, confirmed the homozygous status of the risk haplotype to be unique to the CLP cases. Most of the dogs that were homozygous for the risk allele had blue eyes. Gene annotation and a gene-based association study showed that the risk haplotype encompasses genes implicated in developmental and neurodegenerative disorders. Pathway analysis showed enrichment of glycoproteins and glycosaminoglycans biosynthesis, which play a key role in repairing damaged nerves. In conclusion, our results suggest an important role for the identified candidate region in CLP.

## 1. Introduction

Alaskan sled dogs (ASD), also known as Alaskan Huskies, were originally bred as working dogs for hauling cargo-laden sleds over snow-covered arctic terrain [1]. Over the years, they have evolved as highly aerobic mammals and elite endurance athletes through selective breeding with pure-bred dogs and are used in modern sled dog racing [2,3]. Genetic disorders such as congenital laryngeal paralysis (CLP), which causes respiratory distress, have a debilitating effect on the performance and survival of such elite runners. The earliest incidence of CLP in ASD was reported in the year 1986 [4]. Since then, there has been a paucity of information on the status of CLP in the ASD population in the literature. In contrast, anecdotal evidence through communication with professional mushers in the ASD community suggested CLP to be a commonly known condition, with unknown rates of incidence, and breeders have noted it to be associated with blue eyes and white facial markings [5]. We had previously published a clinical case series on CLP in 25 ASDs and found all CLP cases had blue eyes and white facial markings [5].

Congenital Laryngeal paralysis is an inherited condition in dogs in which one or both recurrent laryngeal nerves are impaired, typically by a degenerative process [6,7,8,9]. This results in the loss of proper functioning of the larynx causing insufficient abduction of the arytenoid cartilages during inspiration resulting in upper airway obstruction [10,11], resulting in breathing difficulties, exercise and heat intolerance, and increased risk of aspiration pneumonia [12]. Respiratory distress, voice impairment (dysphonia), and inspiratory stridor are the main clinical signs of CLP in dogs [5,13]. The affected dogs are known as “Wheezers” within the ASD community due to the abnormal respiratory bruit or wheezing sound commonly made by affected dogs [5]. The degree of respiratory distress is reported to correlate with the degree of nerve impairment and whether the disease is unilateral or bilateral [14]. Diagnosis of CLP is based on clinical signs and is confirmed through laryngeal endoscopic inspection [15]. Affected dogs are surgically treated to improve breathing through unilateral cricoarytenoid lateralization [16,17]. Interestingly, dogs may spontaneously improve with age, however, they are generally unable to become elite-level racing sled dogs [4,5].

Laryngeal paralysis has been reported in several dog breeds, including Alaskan malamutes [18], Bull terriers [19], Bouviers des Flandres [9,20], Siberian huskies [21], Siberian husky x Alaskan malamute crossbreds [7], Leonbergers [22], Dalmatians [23], Labrador retrievers [12], Great Pyrenees [24], and Rottweilers [25,26]. In many of these dogs, laryngeal paralysis is associated with juvenile-onset polyneuropathy, including esophageal dysfunction [13,22,24,25,26]. The combination of laryngeal paralysis and polyneuropathy, also known as laryngeal paralysis and polyneuropathy complex (LPPC), has variable ages of onset [12]. Late-onset forms of LPPC are referred to as geriatric onset laryngeal paralysis polyneuropathy (GOLPP) [27]. Though mutations in genes such as *RAPGEF6* [13], *GJA9* [28], *ARHGEF10* [29], *CNTNAP1* [12], *RAB3GAP1* [30], and *NDRG1* [31] have been found to be associated with canine laryngeal paralysis, they were identified in dogs suffering from polyneuropathy. However, CLP in the Alaskan sled dogs in our study was found to be due to mononeuropathy of the recurrent laryngeal nerves without polyneuropathy [5]. Moreover, the association of several genetic loci, across breeds suggests that mutations causing CLP might be breed specific and complex [12,13,28,29,30,31].

Our aim in this study was to identify the genetic loci associated with mono-neuropathic congenital laryngeal paralysis in ASD.

## 2. Materials and Methods

### 2.1. Sample Collection and Phenotype Assignment

Fifty ASDs were sampled, including 23 male and 27 female dogs. Among these, 20 dogs showed clinical signs of laryngeal paralysis (11 male and 9 female dogs). Since the study was about understanding the genetic basis of congenital laryngeal paralysis (CLP), dogs aged two years or older with no reported breathing problems were designated as controls. Dogs aged less than 1 year that were reported to be suffering from breathing difficulties by owners were clinically examined and confirmed for laryngeal paralysis before inclusion in this study as cases. Dogs that did not meet certain criteria, such as missing medical records, age at first onset, diagnosis with other respiratory disorders, or when clinical signs were observed only after more than 5 miles (8 km) of mushing, were excluded [5]. Physical, neurological, complete blood count, serum biochemical analysis, and orthopedic examinations were performed on all cases. Esophagrams were obtained when possible, using previously described protocols [6].

### 2.2. DNA Isolation and Genotyping

Whole blood samples were collected in 5 mL EDTA tubes. Genomic DNA was isolated from white blood cells using a two-step lysis and salt out method [32]. Each sample’s quantity and quality were checked using a spectrophotometer (Epoch microplate spectrophotometer, USA). Thirty-five samples, including all the cases, were genotyped on an Illumina Canine HD Beadchip containing 173,662 SNPs, while 15 samples were genotyped on a custom Illumina Canine HD chip through Embark Veterinary [33], containing 217,317 single nucleotide polymorphisms (SNPs).

### 2.3. Imputation

Samples genotyped on the SNP chips were merged and filtered for quality using PLINK ver 1.9 [34]. The following thresholds were applied for quality control; SNPs with genotype call rate < 0.90, individual sample call rate < 0.90, minor allele frequency < 0.05, and Hardy Weinberg equilibrium *p* < 1 × 10^−5^ were removed. After quality control, 66,597 SNPs and 50 dogs remained for analysis. Principal component analysis did not show any population stratification, confirming there was no bias due to the choice of the chip used for genotyping. These genotypes were imputed with Minimac4 ver 1.0.2 [35], using a reference panel containing 61,065,811 SNPs from 660 dogs, including ASDs, modern breeds, village dogs, and wild canids [36] (Supplementary File S1). The reference panel was phased on a per-chromosome basis using Beagle ver 5.2 [37]. The reference panel was converted to m3VCF format using minimac3 ver 2.0.1 [35] prior to their use for imputation with minimac4 ver 1.0.2. The target dataset also included 3 dogs for which whole genome sequence data was available. The dataset was then imputed on a per-chromosome basis with default settings. The imputed individual chromosome data were then concatenated using VCFtools 0.1.16 [38] and only bi-allelic SNPs were retained for further analysis. The imputed dataset was tested for imputation accuracy based on genotype concordance and imputation quality score [39]. The variants with a Minimac4 empirical R-squared ≥ 0.6 were retained for downstream analysis and the average genotype concordance rate between imputed and true genotypes was 98.2%. This resulted in a final dataset with 1,054,074 SNPs with an average empirical R-square of 0.96. The SNPs were annotated with the variant effect predictor (VEP) tool [40].

### 2.4. Genome-Wide Association Study

All GWAS conducted in this study were performed using the mixed linear model implemented in GCTA ver 1.91.4 [41]. The model included a genomic relationship matrix estimated with the same genotypes to correct for genomic inflation [42]. Only autosomal SNPs were used in the analysis. Genome-wide significance thresholds were based on Bonferroni correction and were set at 9.487 × 10^−9^ (0.01/1,054,074) for the imputed SNP GWAS, and at 1.50 × 10^−7^ (0.01/66,597) for the original dataset that had 66,597 SNPs. Manhattan plots and QQ-plots were generated with the R package, CMplot [43]. Haplotypes around significantly-associated loci were constructed with Beagle 5.2 [37]. Linkage disequilibrium analysis between SNPs was performed with PLINK ver 1.9 [34]. All the genome positions reported in this study refer to the CanFam3.1 reference assembly (accession number: GCF_000002285.3). The locus zoom plot was generated in R ver 4.2.0 [44] with an open-source script (https://github.com/Geeketics/LocusZooms, accessed on 20 Apirl 2021). Genomic heritability (h^2^) was calculated as the ratio of additive genetic variance (Vg) and phenotypic variance (Vp), and variance components were calculated using the genome-based restricted maximum likelihood (GREML) method implemented in GCTA ver 1.91.4 [41].

### 2.5. Gene-Based Association Study

A gene-based association study using Multi-Marker analysis of genomic annotation (MAGMA) v 1.07 b [45] and the fastBAT option in GCTA ver 1.91.4 [46] was used for identifying potential candidate genes associated with CLP. All SNPs from the GWAS analysis were annotated to genes within 5 kb upstream or downstream, the resulting genes and the summary statistics from the GWAS were used in the gene-based association analysis. The annotation database (CanFam 3.1) was downloaded from Ensembl [40] and included gene location with start and end positions for canine genes. While the fastBAT method leverages linkage disequilibrium and summary level data from GWAS and performs a set-based association analysis, MAGMA uses test statistics for individual SNPs and calculates aggregated *p*-values at the gene level using a known approximation of sampling distributions. KEGG Pathway enrichment analysis of the most significant genes from the gene-based association study (*p* < 0.001) was performed using DAVID [47].

### 2.6. Whole Genome Sequence Analysis

Whole genome sequence data of 3 ASD (2 cases, 1 control) was generated in this study. Illumina TruSeq fragment library with 400 bp inserts were prepared and the libraries were sequenced on a HiSeq 3000 instrument at an average of 20.3 X coverage. A total of 1,730,731,093 reads were generated. The sequence data analysis, variant calling, functional annotation, and prediction of functional effects were performed as described previously [48]. Briefly, the reads were aligned and mapped to the Canine reference assembly, CanFam3.1, using the Burrows-Wheeler Aligner (BWA-mem) ver 0.7.17 [49]. The reads were then sorted by co-ordinates using samtools ver 1.14 [50] and PCR duplicates were mapped with Picard tools ver 2.26.5 (http://broadinstitute.github.io/picard/, accessed on 17 August 2021). Local recalibration, realignment, variant calling, and quality filtering were performed using the Genome analysis tool kit (GATK) ver 4.2 [51]. The SNPs were annotated using SnpEff v5.1 [52]. SNPsift ver 5.1 [52] was used for filtering for non-synonymous variants in the region of interest in the affected dogs. The Integrative Genomics Viewer (IGV) 2.11.6 [53] was used for visual inspection and screening for structural variants in the region of interest in CLP-affected dogs. Sequences generated in this study are deposited in the NCBI database; BioSample ids are given in Supplementary Table S1.

## 3. Results

### 3.1. Phenotype

Clinical presentations, CLP diagnosis, and phenotypes are described in detail by von Pfeil et al., 2018 [5]. The dataset included 20 cases and 30 controls. Blue, brown, and marble-eyed dogs were part of the data set. Marble-eyed dogs are dogs with two or more color in their iris; this condition is referred to as heterochromia iridis. Eye color and sex information for the dogs are given in (Supplementary Table S2). 16 of the 20 cases were blue-eyed, while 20 controls had blue eye color.

### 3.2. Genome-Wide Association Study

**Figure 1:**
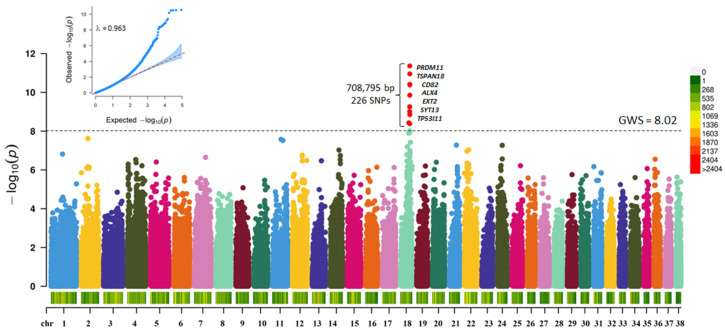
Manhattan plot showing the distribution of *p*-values from the GWAS with imputed SNP data for CLP in ASD. The genome-wide significance was based on Bonferroni corrected *p*-value threshold of 5% (*p* < 9.487 × 10^−9^; −log_10_ *p*-value = 8.02). Red points are SNPs that reached genome-wide significance. The heatmap at the bottom of the plot shows the SNP density. The corresponding quantile-quantile (Q-Q) plot showing the expected −log_10_ *p*-value against the observed −log_10_ *p*-value, is given at the top. The scale color on the right gives the density of SNPs within a window of 1 million bp.

The initial GWAS with 66,597 SNPs (30 Controls and 20 Cases) revealed regions on CFA 18 to be significantly associated with CLP (Supplementary Figure S1 and Table S3). Two SNPs reached Bonferroni corrected genome-wide significance (18: 44849004; *p* < 4.54 × 10^−7^; & 18: 44849276; *p* < 6.8 × 10^−7^). Careful, manual examination of the region around the lead SNP revealed that, on average, there is 1 marker per 18,000 bp, which was ~22% less than the average of 1 marker per 14,450 bp on the Illumina Canine HD chip. Therefore, we performed SNP imputation with a dataset that included the whole genome sequence of 660 dogs [36]. Post quality control of the imputed SNPs, the dataset included 1,054,074 SNPs. A GWAS with the imputed dataset (30 Controls and 20 Cases) revealed a 708,795 bp region on CFA 18 to be significantly associated (*p* < 9.487 × 10^−9^) with CLP (Figure 1, Supplementary Table S4).

**Figure 2:**
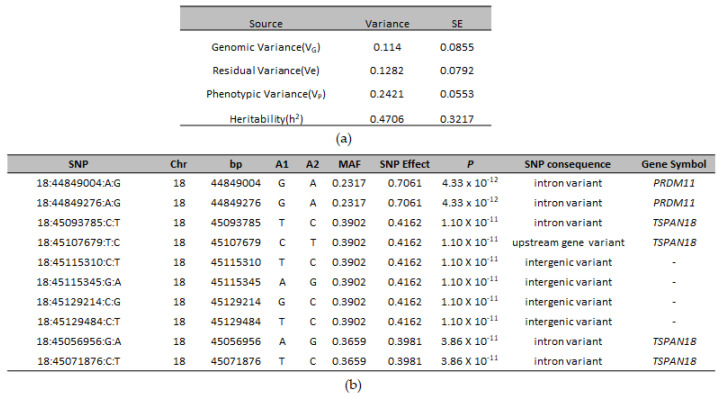
(**a**) Variance component estimates from genomic markers and their respective standard error (s.e.) for CLP in ASD; (**b**) The top ten genome-wide significant SNPs associated with CLP in ASD, their SNP effect, minor allele frequency, association *p*-value, functional consequence, and their annotation with the nearest gene.

Two hundred twenty-six SNPs reached genome-wide significance. The significant SNPs on CFA18 explained 11.4% of the genetic and 24.21% of the phenotypic variance and explained 47.06% of the heritability of CLP in ASD (Figure 2a).

A list of the top genome-wide significantly associated SNPs is given in Figure 2b. The most significant SNP (18:44849004) was an intronic SNP, located in *PRDM11* (PR-Domain Containing Protein 11). Most of the significantly-associated markers were intergenic and intronic variants with low to moderate impact (Supplementary Table S5). However, among these, four markers were missense variants, of which one marker (18:45054697) was predicted to be a deleterious variant (Sift score = 0.04). All four markers were located within *TSPAN18* (Tetraspanin 18) gene. Eighteen of the cases were homozygous for the deleterious variant (Supplementary Table S6). A whole genome sequence analysis of two cases and a control ASD confirmed the presence of this variant in the cases (Supplementary Table S7); however, the functional significance of this variant is unknown.

### 3.3. Linkage Disequilibrium and Associated Haplotypes

**Figure 3:**
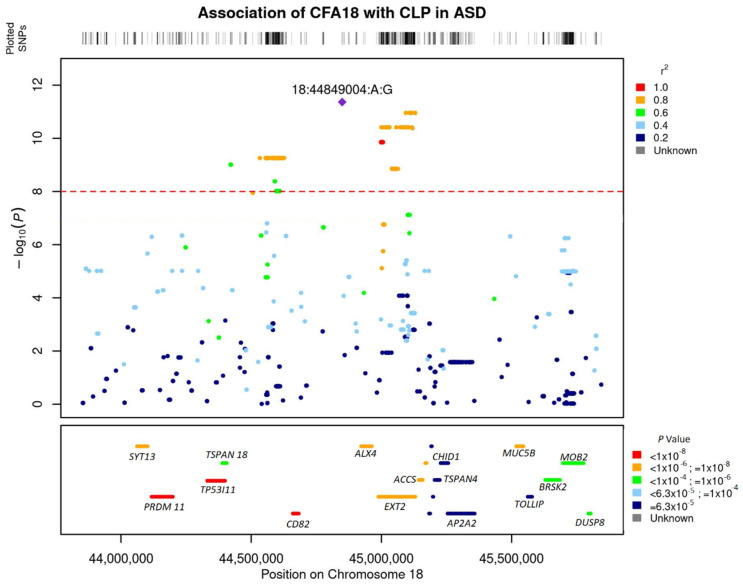
Locus zoom plot showing regional association pattern around lead SNPs on Canine Chromosome 18. The bottom panel shows the layout of genes in the region. The genes are colored based on their enrichment from the gene-based association analysis with GCTA fastbat.

Linkage disequilibrium (LD) analysis revealed a high linkage of the lead SNP with SNPs within 1 MB on CFA 18 (Figure 3). The most significant SNP (18:44849004) was in complete LD (r^2^ = 1) with 18:44849276. These two SNPs were also the most significant SNPs from the GWAS with 66,597 SNP chip data (Supplementary Figure S1). The region of high linkage (r^2^ > 0.6) with the lead SNP encompassed 750 kb, spanning the region 18:44411803–45152544.

GWAS results showed that most of the cases were homozygous for lead SNPs on CFA 18. To define a risk haplotype, we visually inspected the phased haplotypes and performed autozygosity mapping. We searched for a homozygous region with allele sharing among cases around the lead SNP on CFA 18 and found a contiguous haplotype encompassing a region between the two significant SNPs (18:44700683–45132398). The haplotype included 220 SNPs and spanned 431 kb, covering most of the associated significant region in the GWAS analysis (Supplementary Table S6). Eighteen of the 20 cases were homozygous for the risk haplotype, including all the blue and marble-eyed dogs and one brown-eyed dog (Table 1). Among the controls, all the blue-eyed and marble-eyed dogs were heterozygous for the risk haplotype, none of the brown-eyed control dogs carried the haplotype. None of the control dogs were homozygous for the risk haplotype. The risk haplotype overlapped the previously identified duplicated region associated with blue eyes (18:44791417–44890166) [33]. Whole genome sequence analysis confirmed the presence of the duplicated region in both ASD cases, which were blue-eyed. No other structural variants were identified in the region [33]. We then compared the ASD variants with the whole genome sequence of 657 dogs from genetically diverse breeds and found that the risk haplotype was homozygous only in the two ASD cases (Supplementary Table S7).

### 3.4. Annotation of Significant SNPs to Genes and Gene-Based Association Analysis

SNPs were annotated to the nearest genes and the most significant SNPs were within or nearby *PRDM11*, *SYT13* (Synaptotagmin 13), *TP53I11* (Tumor Protein P53 inducible protein 11), *TSPAN18*, *CD82* (Cluster of differentiation 82), *EXT2* (Exostosin Glycosyltransferase 2), *ALX4* (ALX Homeobox 4), *P2RY1* (Purinergic Receptor P2Y1), and *ACCS* (1-Aminocyclopropane-1-Carboxylate Synthase) (Figure 2b, Supplementary Table S8). Gene-based association analysis with MAGMA and fastBAT option in GCTA software identified *PRDM11, SYT13*, *TP53I11*, *ALX4*, *EXT2*, and *TSPAN18* as the most significantly associated genes (*p* < 1 × 10^−6^) based on the number of SNPs that were significantly associated in these genes from the GWAS analysis (Figure 3). Pathway enrichment analysis of the most significant genes showed enrichment for the following terms or pathways; Cholinergic synapse (cfa04725), Glycerolipid metabolism (cfa00561), Glycosaminoglycan biosynthesis, and Glycoprotein (Table 2).

## 4. Discussion

Congenital laryngeal paralysis is an inherited disease that negatively impacts the survival and quality of life of the affected dog. The genetic basis of CLP in ASD has not been explored previously, though the presence of CLP in the ASD has been known for multiple decades. In the present study, we identified a 431 kb haplotype on CFA 18 as a major risk factor for CLP in ASD when in a homozygous state. The risk locus was unambiguously mapped by GWAS. The identified risk haplotype (18:44700683–45132398) overlapped the 98.6 kb duplication (18:44791417–44890166) previously identified to be associated with blue eye color [33].

Several of the affected dogs also had white facial markings. The duplicated region associated with blue eye color in dogs also partially explains facial markings [33]. Association between CLP and blue eyes has been reported in Siberian Husky X Alaskan Malamute crosses and Husky cross breeds [4,7]. In O’Brien and Hendriks’ [4] 1986 report, they mentioned that the CLP condition along with an associated white coat and blue eyes had been known among sled dog owners since the 1960′s. Supporting this association of blue eyes and white facial markings with CLP, the identified risk haplotype encompasses *ALX 4*, which plays an important role in pigmentation [54] and mammalian eye development [55,56]. Several variants within the gene were significantly associated with CLP in the GWAS analysis. All the blue-eyed cases, a brown-eyed case, and a marble-eyed case in our study were homozygous for the risk haplotype, whereas the remaining two brown-eyed cases did not carry the risk haplotype, suggesting that there might be additional genetic factors contributing to CLP. Moreover, the significant SNPs on CFA 18, which included most of the SNPs in the haplotype, explained only 41.06% of the heritability of CLP. However, the location of the major locus for blue eye color within the risk haplotype, and the increased prevalence of the risk haplotype amongst blue-eyed ASD’s, suggests that the causal variant is segregating at a higher frequency amongst blue-eyed ASD’s.

Among the significantly associated genes from the gene-based association study were *EXT2*, *CD82*, *TSPAN18*, *ALX4*, and *TP53I11*. These were among the spectrum of genes found to be involved in Potocki Schaffer syndrome, which affects the development of bones, nerve cells in the brain, and other tissues [57]. *EXT2*, *ALX4*, and *TSPAN18* are expressed in the neuronal crest, which gives rise to the craniofacial skeleton. *MPPED2* (Metallophosphoesterase domain containing 2), located upstream of the risk haplotype, was significantly enriched in the fastbat gene-based association analysis. This gene is thought to play an important role in nervous system development [58]. *MPPED2*, *ALX4*, and *EXT2* are associated with WAGR syndrome (Wilms’ tumor, aniridia, genitourinary anomalies, and mental retardation), a developmental disorder [58,59]. *TSPAN18* plays an important role in the nervous system during cranial neural crest epithelial to mesenchymal transition [60,61]. Mutations in *TSPAN18* have been linked to Schizophrenia [60,62]. *PHF21A* (PHD finger protein 21A), which encodes a histone methyl reader protein (BHC80), regulates a huge number of neuronal genes during embryogenesis [63,64]. Several variants within this gene were found to be significantly associated with CLP in the GWAS analysis and are located upstream of the risk haplotype. *SYT13* (Synaptotagmin 13), a neuroprotective gene located downstream of the risk haplotype, encodes vesicular trafficking proteins that are important for synapsis and vesicle metabolism [65]. Overexpression of *SYT13* was found to preserve motor neurons and delay muscle denervation and improve survival and lifespan in mice affected with amyotrophic lateral sclerosis and spinal muscular atrophy, which are lethal neurodegenerative diseases [66].

Functional enrichment analysis showed the enrichment of glycoproteins and genes involved in glycosaminoglycan biosynthesis. Glycoproteins play a critical role in the upkeep and proper functioning of the nervous system. Axonal glycoproteins are required for nerve polarity routing and repair [67]. In damaged nerves, axonal regeneration is accompanied by the interaction between regenerating neurons and extracellular molecules derived from surrounding neurons or glial cells. Glycosaminoglycans (GAG), which are modified sugar residues, are found in the extracellular matrix and modulate numerous biological processes, such as interactions between proteins by binding to various extracellular molecules, including growth factors and extracellular proteins [68]. GAGs play a critical role in neurodegenerative diseases [68].

The presence of various genes implicated in developmental and neurodegenerative disorders within the risk haplotype, the occurrence of the risk haplotype in a homozygous state in only CLP-affected ASDs (18 out of 20 CLP-affected ASDs), and the absence of the identified haplotype in the genome of 657 dogs from various other breeds not known to be affected with CLP, suggests that the risk haplotype is the putative locus for CLP in ASD.

## 5. Conclusions

In summary, we identified a candidate region on CFA 18 to be associated with congenital laryngeal paralysis in Alaskan sled dogs. Through autozygosity mapping, we narrowed the associated region to a 431 kb haplotype. Our results suggest that the frequency of the risk haplotype is higher amongst ASD with blue eye color. The limitation of our study was the small number of affected dogs and limited pedigree completeness. Many sled dog owners actively breed against blue eyes and white facial markings, and only two additional cases were identified in the past decade. A future follow-on study with a larger number of samples with complete pedigree information is needed to functionally validate the results and identify the causative allele. Despite the limitations, these results provide insights into the genetic basis of CLP in ASD, and the information will be useful for ASD breeding.

## Figures and Tables

**Table 1:** Segregation of the CFA 18:44700683–45132398 risk haplotype associated with CLP in ASD.

		Putative Risk Haplotype (CFA 18:44502007–45132398)		
CLP Status	N	Homozygous Alternative	Heterozygous	Homozygous Risk
Affected	20	2/20	0/20	18/20
Unaffected	205	9/40	21/40	0/40

**Table 2:** Pathway enrichment analysis of the significant genes from the gene-based association analysis.

Term/Pathway	Q-Value ^1^	Genes
Cholinergic synapse	0.002	*GNG4*, *CREB3L1*, *KCNQ1*
Glycosaminoglycan biosynthesis	0.010	*EXT2*, *CHST1*
Glycerolipid metabolism	0.010	*DGKZ*, *PNPLA2*
Glycoprotein	0.010	*EXT2*, *CD82*, *CREB3L1*, *TSPAN18*, *PNPLA2*

^1^ FDR corrected *p*-value.

## Data Availability

Not applicable.

## Supplementary Materials

The following supporting information can be downloaded at: https://www.mdpi.com/article/10.3390/genes13101808/s1, Figure S1: Manhattan plot, showing the distribution of the *p*-values from the GWAS with 66,597 SNPs for CLP in 50 ASD. Points in red are SNPs that were significant. Genome-wide significance was based on Bonferroni corrected *p*-values threshold of 5%. The quantile-quantile (Q-Q) plot on the right shows the expected −log_10_ *p*-value against the observed −log_10_ *p*-value. The scale color on the right of the Manhattan plot gives the density of SNPs within a window of 1 million bp. Table S1: Sample name, breed information, and accession numbers of 660 dogs and their whole genome sequence used in this study; Table S2: Eye color and disease status of samples used in this study; Table S3: Summary statistics from the GWAS with Canine SNP Chip data, the SNPs are ranked based on association *p*-values; Table S4: The 10,000 most significant markers sorted by *p*-value obtained from the GWAS with imputed SNP data; Table S5: Functional annotation information of the most significant SNPs from the GWAS.; Table S6: Haplotype pattern at the identified risk loci on CFA18 in ASD cases and controls; Table S7: Haplotype pattern from WGS data at the identified risk loci on CFA18 in the various dog breeds compared to the ASD cases; Table S8: Summary statistics from the gene-based association analysis with GCTA *fastbat* and MAGMA.
