# Supplementary figures and images for: Genome Wide Association Study with Imputed Whole Genome Sequence Data Identifies a 431 kb Risk Haplotype on CFA18 for Congenital Laryngeal Paralysis in Alaskan Sled Dogs
Source: Genes (Basel). 2022 Oct 6;13(10):1808. doi: 10.3390/genes13101808 (PMC9602090; doi:10.3390/genes13101808)

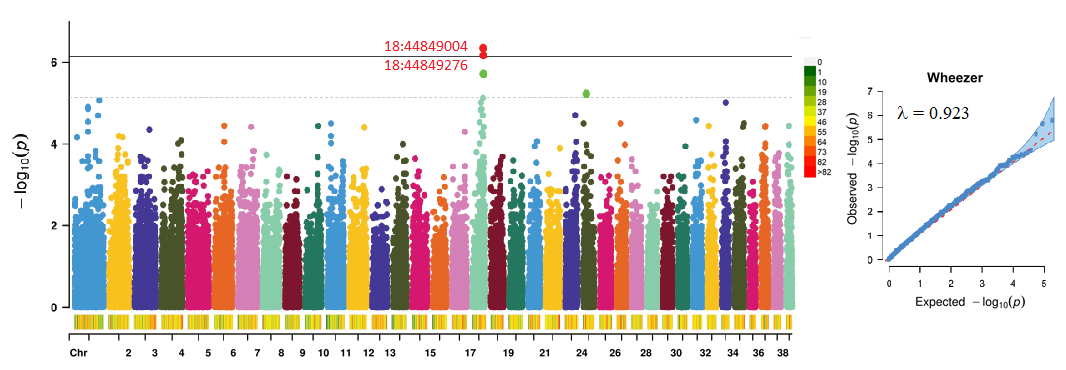

Supplement: Supplementary file 1 [file genes-13-01808-s001.zip › Figure S1.tif]
